# Characterization of Telecare Conversations on Lifestyle Management and Their Relation to Health Care Utilization for Patients with Heart Failure: Mixed Methods Study

**DOI:** 10.2196/46983

**Published:** 2024-10-30

**Authors:** Mojisola Erdt, Sakinah Binte Yusof, Liquan Chai, Siti Umairah Md Salleh, Zhengyuan Liu, Halimah Binte Sarim, Geok Choo Lim, Hazel Lim, Nur Farah Ain Suhaimi, Lin Yulong, Yang Guo, Angela Ng, Sharon Ong, Bryan Peide Choo, Sheldon Lee, Huang Weiliang, Hong Choon Oh, Maria Klara Wolters, Nancy F Chen, Pavitra Krishnaswamy

**Affiliations:** 1 Institute for Infocomm Research (I^2^R) Agency for Science, Technology and Research (A*STAR) Singapore Singapore; 2 School of Informatics University of Edinburgh Edinburgh United Kingdom; 3 Changi General Hospital Singapore Singapore

**Keywords:** telehealth, telecare, heart failure, chronic disease, self-management, lifestyle management, behavior, health care utilization, conversation, dialogue, medical informatics

## Abstract

**Background:**

Telehealth interventions where providers offer support and coaching to patients with chronic conditions such as heart failure (HF) and type 2 diabetes mellitus (T2DM) are effective in improving health outcomes. However, the understanding of the content and structure of these interactions and how they relate to health care utilization remains incomplete.

**Objective:**

This study aimed to characterize the content and structure of telecare conversations on lifestyle management for patients with HF and investigate how these conversations relate to health care utilization.

**Methods:**

We leveraged real-world data from 50 patients with HF enrolled in a postdischarge telehealth program, with the primary intervention comprising a series of telephone calls from nurse telecarers over a 12-month period. For the full cohort, we transcribed 729 English-language calls and annotated conversation topics. For a subcohort (25 patients with both HF and T2DM), we annotated lifestyle management content with fine-grained dialogue acts describing typical conversational structures. For each patient, we identified calls with unusually high ratios of utterances on lifestyle management as lifestyle-focused calls. We further extracted structured data for inpatient admissions from 6 months before to 6 months after the intervention period. First, to understand conversational structures and content of lifestyle-focused calls, we compared the number of utterances, dialogue acts, and symptom attributes in lifestyle-focused calls to those in calls containing but not focused on lifestyle management. Second, to understand the perspectives of nurse telecarers on these calls, we conducted an expert evaluation where 2 nurse telecarers judged levels of concern and follow-up actions for lifestyle-focused and other calls (not focused on lifestyle management content). Finally, we assessed how the number of lifestyle-focused calls relates to the number of admissions, and to the average length of stay per admission.

**Results:**

In comparative analyses, lifestyle-focused calls had significantly fewer utterances (*P*=.01) and more dialogue acts (*P_adj_*=.005) than calls containing but not focused on lifestyle management. Lifestyle-focused calls did not contain deeper discussions on clinical symptoms. These findings indicate that lifestyle-focused calls entail short, intense discussions with greater emphasis on understanding patient experience and coaching than on clinical content. In the expert evaluation, nurse telecarers identified 24.2% (29/120) of calls assessed as concerning enough for follow-up. For these 29 calls, nurse telecarers were more attuned to concerns about symptoms and vitals (19/29, 65.5%) than lifestyle management concerns (4/29, 13.8%). The number of lifestyle-focused calls a patient had was modestly (but not significantly) associated with a lower average length of stay for inpatient admissions (Spearman ρ=-0.30; *P_adj_*=.06), but not with the number of admissions (Spearman ρ=-0.03; *P_adj_*=.84).

**Conclusions:**

Our approach and findings offer novel perspectives on the content, structure, and clinical associations of telehealth conversations on lifestyle management for patients with HF. Hence, our study could inform ways to enhance telehealth programs for self-care management in chronic conditions.

## Introduction

Lifestyle and behavior modifications are widely recognized as cornerstones of effective management of chronic cardiometabolic conditions such as heart failure (HF) or type 2 diabetes mellitus (T2DM). For instance, HF guidelines encourage patients to commit to substantial lifestyle changes involving fluid and dietary restrictions, daily weighing, physical exercise, and adhering to complex medication regimens with regular self-monitoring [1]. Given these elaborate requirements, patients find it difficult to cope with the burden of the disease, its comorbidities, and self-care management challenges. As such, lifestyle management issues persist as unresolved last-mile challenges in cardiometabolic disease management [2-5], which, in turn, lead to potentially avoidable hospitalizations. This is especially evident among patients with HF, whose inpatient costs constitute the largest component of their overall economic burden [6].

In response, numerous education, coaching, and self-management interventions have emerged to support patients in self-care management [7,8]. Increasingly, to enhance access and convenience, these interventions are delivered through telehealth [9-11]. Telehealth interventions for chronic disease management have been evaluated in randomized controlled trials [12,13] and shown to have positive effects on health outcomes and health care utilization [10,14-16].

Commonly, telehealth interventions include nurse-led telecare programs, where trained nurses (telecarers) make regular telephone calls to patients, empowering them with knowledge and skills to independently manage their chronic conditions at home. These telecare conversations serve a central role in the program and involve personalized coaching and self-management support. Such nurse-led telecare programs have proven effective in improving the quality of life and self-care ability of patients with chronic disease in the community [17,18]. However, the communicative content and conversational structure of lifestyle-focused telecare conversations and their relation to health and clinical indicators are not well understood.

Previous works have examined the content of health care conversations using quantitative or qualitative approaches: for example, using visual and correlational analysis to analyze communicative behavior in medical interactions [19,20], applying machine learning to model conversational topic content [21], predicting the patient perception of communication quality [22], and examining narrative arcs over time [23]. Qualitative studies have used thematic coding, ethnography, and conversation analysis to understand clinical conversations [24-26]. However, these studies largely focused on physical encounters and have not assessed the content and structure of lifestyle management exchanges within telecare conversations. Furthermore, related work explores how patient-clinician communication relates to objective or subjective health and clinical indicators [27-29]. For example, increased patient participation in medical decision-making has been linked to improvements in glycated hemoglobin, functional status, and quality of life [30], while physician communication skills have been linked to patient adherence [28]. However, these studies typically considered single, in-person encounters. There has been limited work on telehealth encounters, particularly in chronic disease, where the conversations evolve over time (with the patient’s condition). Addressing these gaps could distill effective structures of communicative behavior that may enhance the design and delivery of telehealth interventions.

Real-world data from routine telehealth delivery offer a substrate to elucidate the content and structure of self-care management conversations and to uncover relations to health and clinical indicators. However, multimodal datasets comprising telehealth conversations and linked medical records are not readily available. Furthermore, addressing such questions requires interfacing quantitative and qualitative analysis of conversational content, communicative behavior, and clinical data, but there has been limited work on such integrative analyses. More multipronged approaches encompassing a mixture of methods could uncover nonapparent patterns of communicative behavior in telehealth settings and their interconnections with health and clinical indicators.

This study aims to characterize the content and structure of telecare conversations on lifestyle management in HF and study how these conversations relate to health care utilization. To this end, we curated a multimodal dataset comprising transcripts of telecare conversations alongside electronic health records for patients with HF enrolled in a postdischarge telehealth program in Singapore. We analyzed this dataset using a mixed methods approach. First, we quantitatively analyzed the communicative content and conversational structures underlying telecarer-patient interactions in lifestyle management calls. Second, we qualitatively assessed telecarer perspectives on how lifestyle management conversations relate to key issues and levels of concern for follow-on clinical actions. Finally, we examined the association between the number of lifestyle-focused calls a patient received and the frequency and length of their inpatient admissions.

## Methods

### HF Telesupport Program

The 12-month HF telesupport program delivers postdischarge support to patients with HF. The intervention was a series of telecarer-patient telephone calls (scheduled and ad hoc) focused on providing personalized education and support. These calls were conducted in English, Mandarin, Malay, or Tamil, or a combination thereof. Each patient received 11 to 15 scheduled calls (based on the patient’s hospital-assessed risk score at enrolment), vital signs telemonitoring, and surveys on symptoms and lifestyle habits. Nurses reviewed patients’ vitals, symptoms, and medical records to determine who needed more urgent support and ad hoc calls. There was a 12-month postintervention follow-up period.

### Study Design

**Figure 1:**
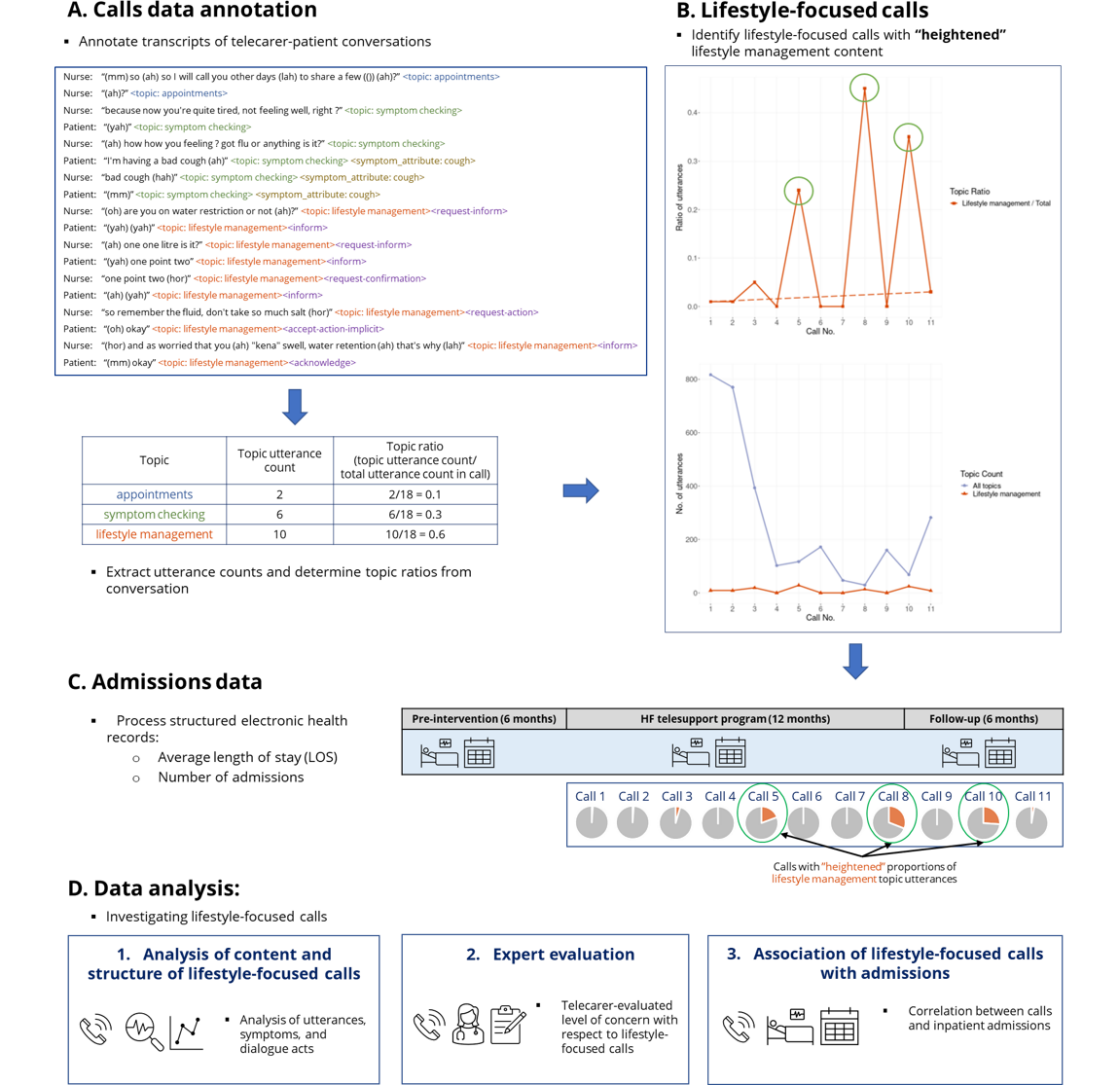
Overview of steps in the study, depicting (A-C) data preparation and (D) analysis of lifestyle-focused calls. HF: heart failure; LOS: length of stay.

Figure 1 illustrates the steps in our study. First, we annotated calls with topics and dialogue acts (Figure 1A). Second, we identified calls with heightened lifestyle management content as lifestyle-focused calls (Figure 1B). Third, we processed structured electronic health records and aligned these with call data (Figure 1C). Subsequently, we investigated the calls from 3 angles (Figure 1D): (1) analysis of the content and conversational structures of lifestyle-focused calls; (2) an expert evaluation of concern levels with respect to lifestyle-focused calls; and (3) the association of lifestyle-focused calls with frequency and length of inpatient admissions. These steps are detailed in the following subsections.

### Data Preparation

#### Overview of Dataset

We extracted inpatient admissions data, recordings of (scheduled and ad hoc) calls (accompanied by telecarers’ task notes), demographics, and medical profiles for this study. For each patient, admissions covered dates from 6 months before and 6 months after the intervention, and calls covered dates from the beginning of the intervention to 6 months after. This timeframe was patient specific. The 6-month postintervention period allowed for admissions data to accrue and to track calls afterward due to varying intervention durations across patients.

In an iterative process, we applied progressive and purposive sampling to select 50 patients with HF (full cohort) with English-language calls from the 150-patient cohort in the original nonrandomized controlled study [14]. Within the specified timeframe, the 50 patients with HF for our retrospective study had 729 English-language, content-driven calls, comprising 117 hours of audio recordings (if multiple calls were made on the same day, eg, due to a faulty connection or separate calls with the caregiver and patient, we counted these as 1 call). We transcribed, deidentified, and manually annotated these 729 calls with topics, symptoms, and attributes. Of these 50 patients, 25 patients had references to T2DM management in their calls (cohort of patients with both HF and T2DM [HFDM]). For these 25 patients (379 calls), we annotated lifestyle management content with dialogue structure information (described below). Finally, from the 25-patient HFDM cohort, we purposively sampled 11 patients who had at least 1 call with lifestyle management content and at least 1 lifestyle-focused call to facilitate a qualitative evaluation (expert evaluation cohort).

#### Calls Data Annotation

We used the Transcriber software (version 1.5.1; developed by Claude Barras and Edouard Geoffrois at DGA in Paris, in collaboration with LDC [University of Pennsylvania]) [31] for transcription and annotation. As the transcribers (SUMS, HL, and NFAS) listened to each audio conversation between telecarers and patients or caregivers, the speakers were manually identified and labeled. Each dialogue was split into utterances. Following convention in computational linguistics and discourse analysis [32,33], we defined utterances as meaningful units of speech delimited by clear pauses. Mostly, utterances correspond to sentences, but they can also correspond to parts of a sentence or a feedback sound such as “mm-hmm.” We preserved the informal and spontaneous styles of spoken interactions, including interlocutor interruption, backchanneling, hesitation, false starts, and repetition [34]. We manually annotated all utterances with the topic of discussion (Table 1).

**Table 1:** Overview of topics, symptoms, attributes, speakers, and dialogue acts. Attributes may fall under any topic. Symptom attributes correspond to respective heart failure symptoms.

Variable		Annotations
**Topics**		Introduction, identification, appointments, telemonitoring, general education, customized coaching, related medical experience, symptom-checking, vitals, medication management, lifestyle management, social-chatting, others
**Symptoms**		Breathlessness, swelling, cough, dizziness, chest pain, heartbeat and palpitations, bleeding, headache
	Symptom attributes	Location, frequency, extent, time, activity
	Lifestyle attributes	Fluid and salt intake, smoking, and alcohol
	Vitals attributes	Blood pressure, weight
	Speakers	Nurse telecarer, patient, caregiver, others
**Dialogue acts**		
	Exchanging information	Request-inform, inform
	Understanding information	Acknowledge, request-clarification, request-confirmation
	Performing action	Request-action, accept-action-implicit, accept-action-explicit, reject-action-implicit, reject-action-explicit
	Evaluation of health condition	Evaluate, evaluate-positive, evaluate-negative
	Social-emotional	Socio-emotional
	Incomplete dialogue act	Back-channel, fragment, stall
	Others	Others

Our study focused on conversation content related to the lifestyle management topic, comprising discussion on adherence to salt and fluid intake restrictions, tobacco and alcohol consumption, and compliance to recommended diet and exercise regimens (Multimedia Appendix 1). Unlike other topics, the lifestyle management topic uniquely focuses on understanding patient experience in relation to lifestyle management.

We annotated lifestyle management utterances (for the HFDM cohort) with dialogue acts to describe dialogue structure. Dialogue acts reflect high-level communication actions that a speaker makes through the utterance, such as exchanging information, understanding information, performing action, evaluation of health condition, or social-emotional utterances [35]. We conducted an interrater reliability test of the dialogue acts annotated by 3 annotators (LC, SUMS, and HL) using Fleiss kappa.

#### Lifestyle-Focused Calls

We identified lifestyle-focused calls to investigate conversation content and communicative behavior relating to the lifestyle management topic. We consider changes in the prevalence of this topic over multiple conversations as suggestive of changes in conversational behavior for each patient, which could possibly indicate corresponding changes in their health and lifestyle management. To identify these changes, we quantified how conversations and behavior evolve over time, using a previously published approach [36,37] that leverages ratios of count and time to quantify behavioral indicators.

Specifically, for each transcribed call (for the full cohort) with lifestyle management content, we calculated utterance ratios for the lifestyle management content within the calls (Figure 1B). We defined an utterance ratio as the utterance count for a topic in a call divided by the total number of utterances in the same call across all topics. This gives a proportion of the utterances made for a specific topic in the call. We defined deviations from the norm as topic-focused calls with heightened content focusing far more on a given topic than the typical call. For each patient, we designated topic-focused calls as those calls fulfilling the criteria: (1) the topic ratio for a call is above the median regression line fitted for each patient’s individual call trajectory, and (2) either the topic ratio for a call is substantially heightened in relation to that of the previous call, or the topic ratio for a call remained heightened following a prior increase from the previous call. Furthermore, 2 authors (ME and SBY) carried out a manual inspection to resolve any ambiguity in distinguishing topic-focused calls from other calls. Notably, to avoid arbitrary thresholds across patients, we highlight that the above process for the selection of topic-focused calls is patient-specific, thereby keeping the quantification of patients’ conversational behavior personalized and within the context of each patient’s calls.

We term calls with heightened lifestyle management content as lifestyle-focused calls and other calls with content relating to the lifestyle management topic, but not to such a heightened extent as calls with lifestyle management content (not focused*)*.

#### Admissions Data

We considered admissions for the full cohort with any primary diagnosis (all-cause inpatient admissions). Elective or planned admissions, such as day surgery, were excluded. We considered the number of admissions and length of stay (LOS, or bed days) in hospital per admission as the 2 indicators for our analyses. These indicators can be considered proxies for the frequency and degree of worsening of a patient’s health. For each patient, we calculated the average LOS per admission by dividing the cumulative LOS over all his or her admissions by his or her total number of admissions within the study timeframe. We could not consider HF-related admissions as the number of such admissions within our dataset was limited.

### Data Analysis

#### Analysis of Content and Structure of Lifestyle-Focused Calls

We comparatively analyzed the number of utterances and dialogue acts in lifestyle-focused calls against those in other calls with lifestyle management content (not focused). As some calls in the 2 groups may come from the same patient, we accounted for this intrapatient correlation using linear regression with standard errors clustered by patient. We corrected for multiple comparisons using Holm-Bonferroni correction. We analyzed symptom attributes, comparing the proportion of lifestyle-focused calls with a mention of symptom attributes to that of calls with lifestyle management content (not focused). We used R software (version 4.3.3; R Foundation for Statistical Computing), including the *fixest* (version 0.12.0) and *stats* (version 4.3.3) packages. We set significance to *P*<.05.

#### Expert Evaluation

To understand how telecarers perceive lifestyle-focused calls, we conducted an expert evaluation study based on the Delphi method [38]. We sought to understand what call contents telecarers find concerning enough to follow up on after a lifestyle-focused call in comparison to other calls (not focused on lifestyle management content). For patients in the expert evaluation cohort, we requested 2 experienced telecarers (HBS and GCL) to review call transcripts in chronological order alongside the patient’s demographic and medical profile and provide their experience-based judgment on which calls they find concerning enough to warrant follow-up actions. We asked them to select their level of concern from predefined follow-up categories already operationalized in the program: business as usual, nurse follow-up: case review, nurse follow-up: call patient, or advise the patient to go to a GP (general practitioner) or polyclinic. Notably, these follow-ups are related to health care utilization, whether actualized or otherwise.

We conducted a pilot study in which 3 patients were studied by the telecarers. Thereafter, the remaining 8 patients were divided equally between the 2 telecarers. We initially designed the study for telecarers to categorize concerns under lifestyle management and medication management. However, during the pilot, the telecarers requested to express additional concerns. Hence, we allowed them to indicate additional concerns as free text remarks. We collated and anonymized the telecarers’ responses. To understand areas where they indicated additional concerns, we analyzed their remarks and other concerns. These were viewed holistically. The resulting categories and assignments of concerns to these categories were based on consensus amongst 2 independent readers (ME and PK) after several rounds of categorization and discussion. We only included concerns resulting clearly from or related to potential issues explicitly discussed in the call. Remarks were only considered when they gave context to telecarers’ concerns and were aligned with the conversation content. We perused the transcripts to ensure no confounds regarding lifestyle management content.

#### Association of Lifestyle-Focused Calls With Admissions

To assess the degree of associations between lifestyle-focused calls and the frequency and length of inpatient admissions, we conducted correlation tests. We computed correlations between the number of lifestyle-focused calls per patient and the number and length (average LOS) of all-cause inpatient admissions. We applied the nonparametric Spearman rank correlation test, as the monotonicity assumption was fulfilled, but normality assumptions could not be guaranteed due to limited dataset size. We corrected for multiple comparisons using Holm-Bonferroni correction. We set significance to *P*<.05. We descriptively compared the number and length of all-cause inpatient admissions between groups of patients with at least 1 lifestyle-focused call and patients with no lifestyle-focused calls. We measured effect size using the Cohen *d* estimate. We used R software, including the *stats* (version 4.3.3) and *effsize* packages (version 0.81).

### Ethical Considerations

This study is set within a Heart Failure Telesupport Program run at the Changi General Hospital (CGH), a 1000-bed acute care tertiary hospital in Singapore. Our retrospective study entailed a secondary analysis of data collected in an earlier nonrandomized controlled study on a 150-patient cohort from January 2014 to October 2017 [14]. The 150-patient cohort in the original study was recruited by trained research coordinators who approached patients eligible for enrolment and obtained written informed consent for their participation in the Heart Failure Telesupport Program and the use of their data for research purposes [14]. As the original nonrandomized controlled study [14] was conducted as part of the follow-up services offered to patients, it was approved by the SingHealth Centralised Institutional Review Board (CIRB) with waiver of ethics review. Our retrospective study was approved by the SingHealth CIRB (Protocol 2018/2761) with waiver of full informed consent. All data used in our retrospective study were deidentified before it was analyzed by the study team.

## Results

### Data Preparation

#### Overview of Dataset

Calls in our dataset involved 50 patients with HF and 8 trained telecarers from the Health Management Unit (HMU) at CGH. The 50 patients had these demographics: 13 (26%) female and 37 (74%) male participants; mean age of 59 (SD 14.3) years; and 29 (58%) Chinese, 8 (16%) Malay, 7 (15%) Indian, and 6 (12%) other participants. The 8 telecarers had these demographics: 1 (12) male and 7 (88%) female participants; mean age of 50.6 (SD 11.1) years; 1 (12) Malay and 7 (88%) Chinese participants; and 2-14 (mean 7.4, SD 4.3) years of experience at CGH [14].

The duration of calls ranged from 33 seconds to 64 minutes 15 seconds (median 7 minutes 30 seconds). Utterances per transcript ranged from 11 to 1289 (median 178). Typically, calls had no or very little lifestyle management content (3584/160,251, 2.24%; Multimedia Appendix 2). The interrater reliability test for the dialogue act annotations resulted in Fleiss κ=0.70. Examples of dialogue acts annotated in the dataset are in Multimedia Appendix 3. Distributions of the frequency and length of inpatient admissions are in Multimedia Appendix 4.

#### Cohort Characteristics

The 3 patient cohorts generally have comparable demographics, with patients being predominantly male, of Chinese ethnicity, and aged between 50 and 70 years (Table 2). Patients had, on average, 15 to 16 calls, with very few (2 to 3) lifestyle-focused calls. The expert evaluation cohort, however, due to its small size and having been purposively sampled to facilitate a qualitative evaluation, had very few (3/11, 27%) female patients and slightly more calls (mean 16.3, SD 4.7) and lifestyle-focused calls (mean 3.4, SD 1.3). On average, patients had 3 to 4 all-cause inpatient admissions, with an average LOS of about 6 days; this was comparable across cohorts. The cohorts were also comparable regarding the Charlson Comorbidity Index (CCI), the New York Heart Association (NYHA) classes, and medication classes (Multimedia Appendix 5).

**Table 2:** Characteristics of the full cohort (50 patients with HF^a^), HFDM^b^ cohort (25 patients with both HF and T2DM^c^), and expert evaluation cohort (11 patients with both HF and T2DM, a subset of the HFDM cohort).

		Full cohort (N=50)^d^	HFDM cohort (n=25)^d^	Expert evaluation cohort (n=11)^d^
**Age in years, mean (SD)**		59 (14.3)	58.3 (12.8)	61.1 (12)
**Gender, n (%)**				
	Male	37 (74)	21 (84)	8 (73)
	Female	13 (26)	4 (16)	3 (27)
**Ethnicity, n (%)**				
	Chinese	29 (58)	13 (52)	5 (45)
	Malay	8 (16)	5 (20)	2 (18)
	Indian	7 (14)	3 (12)	3 (27)
	Others	6 (12)	4 (16)	1 (9)
**Number of inpatient admissions, mean (SD)**				
	All-cause	3.4 (2.8)	3.8 (2.4)	3.3 (2.6)
	CVD^e^-related	2.0 (2.1)	1.8 (1.1)	1.4 (0.5)
	HF-related	1.8 (1.3)	1.8 (1.4)	1.4 (0.8)
**Average LOS** ^f^ **, mean (SD)**				
	All-cause	6.5 (2.4)	6.1 (2.6)	5.7 (2.6)
	CVD-related	6.5 (2.3)	6.0 (1.9)	5.9 (1.9)
	HF-related	6.6 (2.6)	5.9 (2.4)	5.7 (2.8)
**Calls, mean (SD)**				
	All calls	14.6 (7.1)	15.2 (7)	16.3 (4.7)
	Lifestyle-focused calls	2.0 (1.9)	2.5 (1.9)	3.4 (1.3)
	Calls with lifestyle management content (not focused)	2.8 (3.6)	3.7 (4.3)	3.5 (2.7)
	Calls without lifestyle management content	9.8 (8.1)	9.0 (7.9)	9.4 (6.3)

^a^HF: heart failure.

^b^HFDM: both heart failure and type 2 diabetes mellitus.

^c^T2DM: type 2 diabetes mellitus.

^d^For some indicators, data may not be available for all patients.

^e^CVD: cardiovascular disease.

^f^LOS: length of stay.

### Data Analysis

#### Analysis of Content and Structure of Lifestyle-Focused Calls

To give an impression of the content and conversation structures in lifestyle-focused calls, we present 2 excerpts of conversation with dialogue act annotations. In the first example (Multimedia Appendix 6), the patient is feeling unwell, and the telecarer checks on his general condition and discusses different aspects of HF lifestyle management. Request-inform and inform are used to check for compliance with fluid restriction and to educate the patient. Request-action is used to reinforce correct adherence behavior, although this takes up a small part of the conversation. The second example (Multimedia Appendix 7) demonstrates how socio-emotional dialogue acts are used for relationship-building and expressing emotions. Evaluate-negative indicates where the telecarer highlights an issue with the patient’s condition, while inform provides background regarding the patient’s condition and approach. This call shows the difficulty in establishing the patient’s actual actions relating to lifestyle management and highlights the shared burden of care between the caregiver and telecarer in helping to manage the patient’s condition.

We now present the results of the analyses. First, for the full cohort, we find that lifestyle-focused calls had significantly fewer utterances (mean 203.1, SD 147.3) than calls with lifestyle management content (not focused) (mean 310.5, SD 217.1; *P*=.01). Lifestyle-focused calls are therefore shorter than calls with lifestyle content (not focused) in terms of number of utterances. Furthermore, the proportion of lifestyle-focused calls with a mention of symptom attributes is comparable with that of calls with lifestyle management content (not focused) (Multimedia Appendix 8).

Next, we see significantly more dialogue acts on average in lifestyle-focused calls (mean 17.5, SD 12.4) than in calls with lifestyle management content (not focused; mean 10.1, SD 7.7; *P_adj_*=.005; Table 3). We observe similar trends across categories of dialogue acts: exchanging information (mean 10.8, SD 7.8 versus mean 7.0, SD 5.2; *P_adj_*=.01), understanding information (mean 3.0, SD 2.8 versus mean 2.0, SD 2.0; *P_adj_*=.04), and incomplete dialogue acts (mean 1.4, SD 2.4 versus mean 0.4, SD 0.8; *P_adj_*=.01). As dialogue act annotations were only available for the 25-patient HFDM cohort, we focused these analyses on them.

**Table 3:** Linear regression results comparing dialogue acts, grouped by category, for lifestyle-focused calls (n=49) and calls with lifestyle management content (not focused; n=80). Multiple dialogue acts may be annotated within a single utterance. SEs clustered by patient, with *P*<.05, and Holm-Bonferroni correction for multiple comparisons.

	Lifestyle-focused calls (n=49)				Calls with lifestyle management content (not focused) (n=80)				Linear regression with clustered SEs^a^			
	Dialogue acts, n	Mean (SD)	Median (IQR)	Dialogue acts, n		Mean (SD)	Median (IQR)	Estimate		95% CI	*P* value	*P*_*adj*_ value^b^
Overall dialogue acts	857	17.5 (12.4)	15.0 (9.0-24.0)	807		10.1 (7.7)	8.0 (5.0-13.0)	7.4		3.8 to 11.0	<.001	.005
Exchanging information	531	10.8 (7.8)	10.0 (5.0-16.0)	557		7.0 (5.2)	6.0 (3.0-8.0)	3.9		1.7 to 6.0	<.001	.01
Understanding information	146	3.0 (2.8)	2.0 (1.0-4.0)	154		2.0 (2.0)	1.0 (0.0-3.0)	1.1		0.3 to 1.8	<.001	.04
Performing action	39	0.8 (1.4)	0.0 (1.0-2.0)	15		0.2 (0.7)	0.0 (0.0-0.0)	0.6		0.1 to 1.2	.03	.12
Evaluation of health condition	21	0.4 (1.0)	0.0 (0.0-0.0)	16		0.2 (0.7)	0.0 (0.0-0.0)	0.2		–0.2 to 0.6	.24	.40
Social-emotional	29	0.6 (1.7)	0.0 (0.0-0.0)	15		0.2 (0.6)	0.0 (0.0-0.0)	0.4		–0.2 to 1.0	.17	.40
Incomplete dialogue act	68	1.4 (2.4)	1.0 (0.0-2.0)	30		0.4 (0.8)	0.0 (0.0-0.3)	1.0		0.4 to 1.6	<.001	.01
Others	25	0.5 (0.8)	0.0 (0.0-1.0)	21		0.3 (0.9)	0.0 (0.0-0.0)	0.2		–0.1 to 0.6	.13	.40

^a^Lifestyle-focused call (versus call with lifestyle management content [not focused]).

^b^Holm-Bonferroni correction.

Overall, while lifestyle-focused calls had fewer utterances than other calls with lifestyle management content (not focused), the discussions in lifestyle-focused calls were more intense with more dialogue acts. In particular, the higher numbers of exchanging and understanding information dialogue acts suggest that lifestyle-focused calls have more probing and coaching exchanges. Conversely, discussions on symptoms were comparable across both types of calls, suggesting that clinical topics did not have greater attention in lifestyle-focused calls.

#### Expert Evaluation

The telecarers did not flag many calls as concerning (Table 4). Only 24.2% (29/120) of the calls were found concerning enough to follow up on. Of the 30 lifestyle-focused calls among the 120 calls, 30% (9/30) were flagged with at least 1 concern. We found that the telecarers had concerns in 8 areas: 1-HF symptoms (eg, swelling, bloated stomach, cough, chest pain, and shortness of breath); 2-Influenza symptoms (eg, fever, flu, viral infection, and runny nose); 3-Gastrointestinal symptoms (eg, stomach discomfort, abdominal pain, constipation, and diarrhea); 4-Vitals (eg, weight gain, blood pressure, and weight loss); 5-Comorbidities (eg, cancer and diabetes); 6-Lifestyle management (eg, fluid and salt intake); 7-Medication management (eg, medication adjustment, self-administration, and adherence); and 8-Others (eg, dental issues leading to weight loss).

In terms of areas of concern, telecarers were more attuned to concerns related to 1-HF symptoms (12/120, 10%) and 4-Vitals (12/120, 10%), with 5 calls related to both, rather than to concerns related to 6-Lifestyle management (4/120, 3.3%). Even within lifestyle-focused calls, only 2 of the calls were flagged with concerns related to 6-Lifestyle management.

**Table 4:** Areas of concern identified by the telecarers, their level of concern, and the proportion of calls with concerns across all calls (n=120) and across lifestyle-focused calls (n=30).

		Areas of concern identified by telecarers:									Calls^a^ with at least 1 concern		Calls^a^ with no concern
		1-HF^b^ symptoms	2-Influenza symptoms	3-Gastrointestinal symptoms	4-Vitals	5-Comorbidities	6-Lifestyle management	7-Medication management	8-Others				
**All calls**													
	Business as usual, n	1	0	0	2	0	0	1	1	—c		—	
	Nurse follow-up: case review, n	6	4	2	4	4	4	2	0	—		—	
	Nurse follow-up: call patient, n	1	0	0	3	3	0	0	0	—		—	
	Advise patient to go to GP^d^ or polyclinic, n	4	2	1	3	0	0	0	0	—		—	
	Total number of calls^a^, n (%)	12 (10.0)	6 (5.0)	3 (2.5)	12 (10.0)	7 (5.8)	4 (3.3)	3 (2.5)	1 (0.8)	29 (24.2)		91 (75.8)	
**Lifestyle-focused calls**													
	Business as usual, n	0	0	0	0	0	0	1	0				
	Nurse follow-up: case review, n	1	1	1	1	1	2	0	0				
	Nurse follow-up: call patient, n	1	0	0	1	0	0	0	0				
	Advise patient to go to GP or polyclinic, n	3	0	0	1	0	0	0	0				
	Total number of calls^a^, n (%)	5 (16.7)	1 (3.3)	1 (3.3)	3 (10.0)	1 (3.3)	2 (6.7)	1 (3.3)	0	9 (30)		21 (70)	

^a^Calls may have overlapping concerns.

^b^HF: heart failure.

^c^Not applicable.

^d^GP: general practitioner.

The 4 calls flagged with concerns related to 6-Lifestyle management (including 2 lifestyle-focused calls) were all accompanied by concerns relating to 4-Vitals; 1 of the lifestyle-focused calls additionally had a concern relating to 1-HF symptoms. The telecarers assigned the level of concern “Nurse follow-up: case review” to all 4 of these calls.

In summary, the expert evaluation shows that telecarers do not often flag calls as concerning enough to follow up on beyond the call. Concerns, when flagged, primarily focus on HF symptoms and vitals (19/29, 65.5%). The few calls flagged with lifestyle management concerns (4/29, 13.8%) were consistently accompanied by concerns relating to other clinical issues and followed up via case review. As such, telecarers do not typically find lifestyle-focused calls any more concerning than other calls (not focused on lifestyle management content).

#### Association of Lifestyle-Focused Calls with Admissions

**Figure 2:**
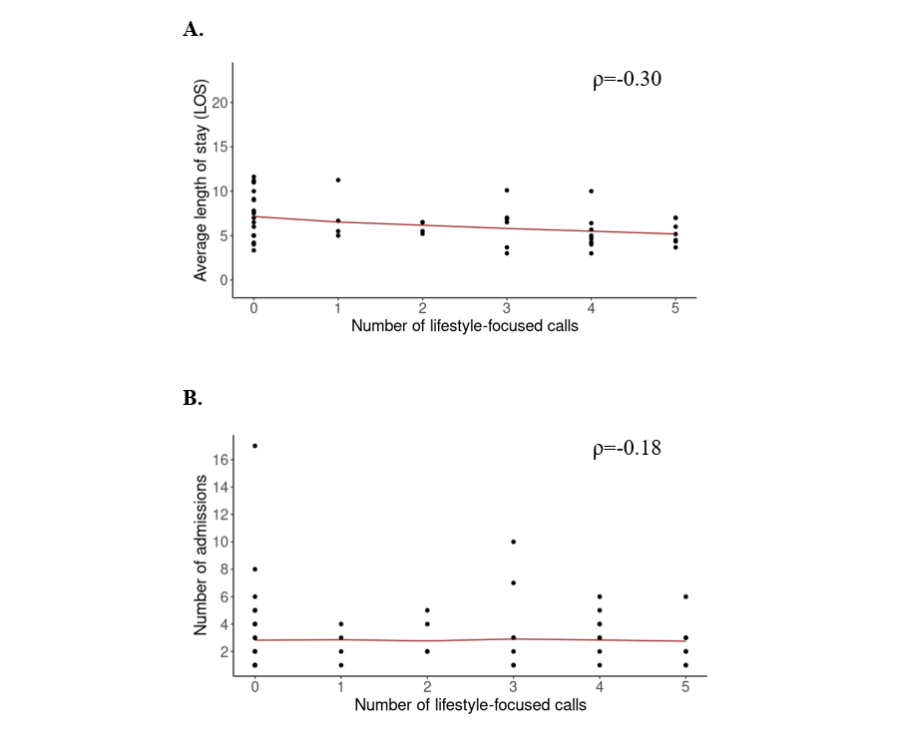
Correlation between the number of lifestyle-focused calls and (A) average length of stay (LOS) for all-cause inpatient admissions and (B) number of all-cause inpatient admissions.

We present results of the association between the number of lifestyle-focused calls and (1) average LOS for all-cause inpatient admissions and (2) number of all-cause inpatient admissions in Figure 2. Each data point corresponds to 1 patient. Black dots represent the average LOS and number of admissions per patient, respectively. Trends in average LOS and number of admissions are represented with smoothed lines fitting locally weighted regression curves (LOWESS; N=50). The number of lifestyle-focused calls has a modest negative correlation with average LOS for all-cause inpatient admissions (ρ=–0.30; *P*=.03; *P_adj_*=.06) (Figure 2A), but a negligible negative correlation with the number of all-cause inpatient admissions (ρ=-0.18; *P*=.84; *P_adj_*=.84; Figure 2B). Both findings are not statistically significant after accounting for multiple comparisons.

We further compared the frequency and length of admissions for patients with no lifestyle-focused calls (20/50, 40%) versus for patients with at least 1 lifestyle-focused call (30/50, 60%; Multimedia Appendix 9). Patients with at least 1 lifestyle-focused call have lower average LOS on average (mean 3.2, SD 2.1) compared to patients with no lifestyle-focused calls (mean 4.0, SD 1.4), with a medium effect size (Cohen *d*=–0.62). Similarly, patients with at least 1 lifestyle-focused call have slightly fewer average admissions (mean 3.2, SD 2.1) compared to patients with no lifestyle-focused calls (mean 4.0, SD 1.4), with a small effect size (Cohen *d*=–0.21).

We considered whether the association of lifestyle-focused calls to average LOS could be attributed to a higher number of symptoms mentioned in the calls (Multimedia Appendix 8). Lifestyle-focused calls have fewer to similar proportions of symptom attributes as compared to calls with lifestyle management content (not focused).

Overall, these findings suggest that having more calls focused on discussing lifestyle management may be related to shorter (but not fewer) inpatient stays.

## Discussion

### Principal Findings

Nurse-led telehealth interventions improve the self-care behaviors of patients with chronic cardiometabolic conditions, but the content, structure, and clinical associations of lifestyle-focused telecarer-patient conversations are not well understood. We leveraged a real-world multimodal dataset with a unique mixed methods approach to elucidate novel insights on telecarer-patient conversations focused on lifestyle management. First, we found that compared to typical calls with lifestyle management content, calls with a greater focus on lifestyle management were shorter and more intense but did not emphasize discussion of symptoms. Furthermore, we found that experienced telecarers are more attuned to symptoms and vitals as causes for concern than to lifestyle management. Finally, we found that a greater focus on lifestyle management in telecare conversations is associated with modest but nonsignificant drops in the extent of inpatient care required.

### Implications

Our quantitative analyses showed that lifestyle-focused calls entail conversational structures reflective of coaching activities informed by an understanding of patient experiences on behavioral or lifestyle changes [39]. Such coaching activities are beneficial to patient participation and lifestyle management for chronic disease [12,17,20]. Notably, while previous studies have studied communication in telehealth programs as a whole [26,39,40], we zoomed in on conversations where telecarers prioritized lifestyle management (perhaps in response to changes in patient experience or behavior) and conducted rigorous linguistic analyses (spanning utterances, dialogue acts, and attributes) on the underlying conversational structures. Hence, our work provides targeted insights into content and structures of conversations promoting lifestyle management in telehealth settings and could inform enhancements to telecare programs.

That said, our qualitative evaluations revealed the inherent challenges of addressing lifestyle management issues in telehealth settings. From the telecarers’ perspective, lifestyle management issues were rarely concerning enough to follow up on, partly because they assessed that any lifestyle management issues that arose during calls were (to the extent possible) addressed and reinforced within the calls themselves. Indeed, with time constraints and increasing numbers of patients, telecarers may often grapple with the contention of addressing acute needs arising from symptom checking and vitals monitoring versus investing in long-term gains from lifestyle management. Hence, supplementing calls with integrated care processes or digital solutions could enable more resource-efficient means to detect and follow up on lifestyle management issues.

Our association analyses showed that a greater focus on intensive lifestyle management support in telecarer-patient conversations had a discernable (but nonsignificant) association with the average length of inpatient stays but not with the number of admissions. While it is known that nurse-led telecoaching improves patients’ HF-related self-care behaviors [10,41], our study is notable for suggesting possible links between lifestyle-focused telecare conversations and health care utilization. Intuitively, one might expect that patients with more lifestyle-focused calls would be more symptomatic and, hence, have longer hospital stays. However, our results point to a different possibility: that having more calls focusing on lifestyle management issues may be linked to the increased likelihood of such issues being promptly addressed; that is, these calls could correspond to timely interventions that reinforce positive behaviors and mitigate future progression to severe admissions. The potential effectiveness of these lifestyle-focused interventions possibly also explains the lack of associations with the frequency of inpatient admissions [14,17,18]. That said, the associations are modest, possibly as the length of inpatient stay is influenced by a wide range of complex factors [27,42,43], and telehealth conversation contents are only indirect indicators of clinical state [44].

Collectively, our findings motivate the design of processes and solutions to enable telecarers to effectively address longer-term lifestyle management issues alongside acute clinical priorities for HF and other cardiometabolic conditions. For example, digital tools could regularly bring telecarers’ attention to lifestyle management issues, thereby enabling timely and focused coaching and reinforcement in calls. Furthermore, the inclusion of systematic follow-up actions that telecarers could suggest to patients experiencing lifestyle management issues beyond typical follow-ups for symptoms and clinical issues may be beneficial.

### Strengths and Limitations

A notable strength of our study is the multimodal dataset linking unstructured telehealth conversations with structured electronic health records. Another strength is the analysis approach leveraging behavioral science–driven strategies [36,37] to assess changes in conversational behavior over time, as this allowed us to pinpoint calls where telecarers accorded heightened priority to lifestyle management. Further strengths include the rigorous linguistic analyses of these calls using fine-grained dialogue acts, the expert evaluation, and the associations of calls with admissions, as these together provide a holistic picture of interfacing behavioral, conversational, and clinical considerations for new insights.

Resource constraints for data curation led to some limitations. Our dataset was limited to English conversations for only 50 patients, and potential patient selection bias should be noted. Singapore is a multi-ethnic society in Southeast Asia, with many residents being conversant in at least 2 languages (English, Mandarin, Malay, or Tamil). However, fluency in English could possibly correlate to higher education and socioeconomic status. Furthermore, for efficient annotation, only a single topic was annotated per utterance, but this was mitigated by assigning the predominant topic to each utterance. We also could not perform qualitative coding and analysis of the call transcripts, but future efforts in this direction could provide more nuanced findings. Furthermore, the paucity and irregularity of admissions data across patients introduced challenges for association analyses. While we mitigated these by adopting a patient-specific timeframe, limitations in dataset size curtailed granular analyses (eg, of HF-related admissions) and statistical power. Future efforts with larger cohorts could be valuable.

### Conclusion

This study provides new perspectives on the content and structure of telecare conversations and highlights that a focus on lifestyle management could play an important role in improving the care of patients with HF. As such, our findings offer the potential to inform ways to enhance fast-proliferating telehealth programs for improved self-care in chronic conditions.

## Appendix

Multimedia Appendix 1 Description of topics, symptoms, attributes, speakers, and dialogue acts annotated in the dataset. Symptoms, lifestyle attributes, and vitals attributes may fall under any of the topics listed. Symptom attributes correspond to the respective heart failure symptoms.

Multimedia Appendix 2 Proportions of utterances annotated in the dataset (n=160,251) across topics and speakers. Proportions of symptoms (n=35,114), symptom attributes (n=1036), lifestyle attributes (n=4992), and vitals attributes (n=45,474) annotated in the dataset. Symptom attributes (location, frequency, extent, time, and activity) correspond to the respective heart failure symptoms.

Multimedia Appendix 3 Examples of dialogue acts annotated in the dataset.

Multimedia Appendix 4 Distributions (histograms) of (A) the number of inpatient admissions and (B) average length of stay (LOS) per patient, for all-cause inpatient admissions (50 patients). Vertical dotted lines indicate mean values for number of inpatient admissions and average LOS.

Multimedia Appendix 5 Characteristics of the full cohort (50 patients with HF), HFDM cohort (25 patients with both HF and T2DM), and expert evaluation cohort (11 patients with both HF and T2DM, a subset of the HFDM cohort).

Multimedia Appendix 6 Excerpt of conversation on lifestyle management between patient P05 and nurse telecarer N02.

Multimedia Appendix 7 Excerpt of conversation on lifestyle management between patient P03’s spouse and nurse telecarer N06.

Multimedia Appendix 8 Comparison of proportions of lifestyle-focused calls (n=100) and calls with lifestyle management content (not focused; n=138) that include mentions of symptom attributes.

Multimedia Appendix 9 Comparative analysis of the average length of stay (LOS) for all-cause inpatient admissions and number of all-cause inpatient admissions between patients with at least 1 lifestyle-focused call (n=30) and patients with no lifestyle-focused calls (n=20).

## Abbreviations

CCI: Charlson Comorbidity Index
CGH: Changi General Hospital
CIRB: Centralised Institutional Review Board
GP: general practitioner
HF: heart failure
HFDM: heart failure and type 2 diabetes mellitus
HMU: Health Management Unit
LOS: length of stay
NYHA: New York Heart Association
T2DM: type 2 diabetes mellitus

## Data Availability

The datasets generated during and/or analyzed during this study are not publicly available due to the need to protect patient privacy but may be made available on reasonable request to Dr. Oh Hong Choon (oh.hong.choon@singhealth.com.sg), under restrictions subject to obtaining ethics approval from the institutional review board(s) and an appropriate data-use and/or research agreement with Changi General Hospital.
