# Supplementary material for: Characterization of Telecare Conversations on Lifestyle Management and Their Relation to Health Care Utilization for Patients with Heart Failure: Mixed Methods Study
Source: J Med Internet Res. 2024 Oct 30;26:e46983. doi: 10.2196/46983 (PMC11561433; doi:10.2196/46983)
Supplement: Multimedia Appendix 1 [file jmir_v26i1e46983_app1.docx]

**Multimedia Appendix 1**

Multimedia Appendix 1 (Table). Description of topics, symptoms, attributes, speakers, and dialogue acts annotated in the dataset. Symptoms, lifestyle attributes, and vitals attributes may fall under any of the topics listed. Symptom attributes correspond to the respective HF symptoms under symptoms.

| Annotations | | Description |
| --- | --- | --- |
| **Topics** | | |
|  | Introduction | Salutations and greetings |
|  | Identification | Checks on patient’s IC, address, and date of birth |
|  | Appointments | Discussion of past and future HF appointments |
|  | Telemonitoring | Discussion on telemonitoring program and patient’s self-monitoring of vitals |
|  | General education | General HF-related teaching to all patients with HF |
|  | Customized coaching | HF-related self-care teaching tailored to patient's needs |
|  | Related medical experience | Discussion on non-HF related health issues and medication, comorbidities, and past HF episodes prior to telemonitoring program |
|  | Symptom-checking | Checks on HF symptoms |
|  | Vitals | Discussion on patient's telemonitored vitals |
|  | Medication management | Discussion on HF medication |
|  | Lifestyle management | Checks on compliance to fluid and salt restriction, tobacco use, and alcohol consumption |
|  | Social chatting | Relationship building between nurse telecarer and patient |
|  | Others | Other topics discussed |
| **Symptoms** | | |
|  | Breathlessness | Discussion on the HF symptom breathlessness |
|  | Swelling | Discussion on the HF symptom swelling |
|  | Cough | Discussion on the HF symptom cough |
|  | Dizziness | Discussion on the HF symptom dizziness |
|  | Chest pain | Discussion on the HF symptom chest pain |
|  | Heartbeat and palpitation | Discussion on the HF symptom heartbeat and palpitation |
|  | Bleeding | Discussion on the HF symptom bleeding |
|  | Headache | Discussion on the HF symptom headache |
| **Symptom attributes** | | |
|  | Location | Discussion on the location of a HF symptom (e.g., “tummy”, “toe all the ten”) |
|  | Frequency | Discussion on the frequency of occurrence of a HF symptom e.g., “sometimes”, “on and off”, “always there”, “it comes it goes”, “once in a while” |
|  | Extent | Discussion on the extent of a HF symptom (e.g., “not too much”, “a bit bloated”, “phlegm”, “a bit of discomfort”, “a bit better”, “dry”) |
|  | Time | Discussion on the time of occurrence of a HF symptom (e.g., “day and night”, “daytime also cough”, “depends”) |
|  | Activity | Discussion on the activity when there is an occurrence of a HF symptom (e.g., “if there are many people around”, “fever”, “when I am awake doing nothing, normal”) |
| **Lifestyle attributes** | | |
|  | Fluid and salt intake | Discussion on the intake of fluid or salt |
|  | Smoking and alcohol | Discussion on smoking and alcohol consumption |
| **Vitals attributes** | | |
|  | Blood pressure | Discussion on blood pressure |
|  | Weight | Discussion on weight |
| **Speakers** | | |
|  | Nurse telecarer | Speaker is a nurse telecarer |
|  | Patient | Speaker is a patient enrolled in the Heart Failure Telesupport Program |
|  | Caregiver | Speaker is the patient’s caregiver, e.g., spouse, daughter, son, daughter-in-law, son-in-law |
|  | Others | Speaker is a coordinator or administrator in the Heart Failure Telesupport Program |
| **Dialogue acts** | | |
|  | Exchanging information | When a speaker attempts to provide or ask for a new piece of information from the hearer. Usually expressed in the form of a question. Comprises: request-inform, inform. |
|  | Understanding information | When the speaker explicitly acknowledges to show he/she understands or tries to get clarification about the piece of information provided by the hearer. Comprises: acknowledge, request-clarification, request-confirmation. |
|  | Performing action | When the speaker describes a specific action for the hearer to conduct, not to conduct, or stop to conduct that action. The hearer will explicitly/implicitly agree/disagree to perform the requested action.  Comprises: request-action, accept-action-implicit, accept-action-explicit, reject-action-implicit, reject-action-explicit. |
|  | Evaluation of health condition | When the nurse telecarer makes comments and judgements to express thoughts about whether the patient’s current daily life situation and health condition is good or bad. Or when the nurse telecarer tries to assess how well the patient has done or assess the knowledge that the patient has.  Comprises: evaluate, evaluate-positive, evaluate-negative. |
|  | Social emotional | When the speaker attempts to express an utterance which relates to emotion to build rapport with the hearer.  Comprises: socio-emotional. |
|  | Incomplete dialogue act | Utterances that do not convey full intentions.  Comprises: back-channel, fragment, stall. |
|  | Others | When the speaker conveys a full intention, but it does not fit into any of the other labels. E.g., utterances that do not contribute to the purpose of the conversation. |
