# Supplementary material for: Characterization of Telecare Conversations on Lifestyle Management and Their Relation to Health Care Utilization for Patients with Heart Failure: Mixed Methods Study
Source: J Med Internet Res. 2024 Oct 30;26:e46983. doi: 10.2196/46983 (PMC11561433; doi:10.2196/46983)
Supplement: Multimedia Appendix 2 [file jmir_v26i1e46983_app2.docx]

**Multimedia Appendix 2**

Multimedia Appendix 2 (Table). Proportions of utterances annotated in the dataset (N=160,251) across topics and speakers. Proportions of symptoms (N=35,114), symptom attributes (N=1036), lifestyle attributes (N=4992), and vitals attributes (N=45,474) annotated in the dataset. Symptom attributes (location, frequency, extent, time, activity) correspond to the respective HF symptoms.

| **Annotations** | | **Number of utterances or attributes** |
| --- | --- | --- |
| **Topics, n (%)** | | |
|  | Telemonitoring | 35,306 (22.03) |
|  | Related medical experience | 21,796 (13.60) |
|  | General education | 19,623 (12.25) |
|  | Symptom checking | 17,927 (11.19) |
|  | Customized coaching | 12,771 (7.97) |
|  | Medication management | 11,830 (7.38) |
|  | Appointments | 8220 (5.13) |
|  | Social chatting | 7657 (4.78) |
|  | Introduction | 6752 (4.21) |
|  | Vitals | 5458 (3.41) |
|  | Identification | 4657 (2.91) |
|  | Lifestyle management | 3584 (2.24) |
|  | Others | 5667 (3.54) |
| **Symptoms, n (%)** | |  |
|  | Breathlessness | 8454 (24.08) |
|  | Swelling | 7179 (20.44) |
|  | Cough | 6161 (17.55) |
|  | Dizziness | 5379 (15.32) |
|  | Chest pain | 3866 (11.01) |
|  | Heartbeat and palpitation | 2936 (8.36) |
|  | Bleeding | 646 (1.84) |
|  | Headache | 493 (1.40) |
| **Symptom attributes, n (%)** | | |
|  | Breathlessness attributes | 163 (15.73) |
|  | Swelling attributes | 262 (25.29) |
|  | Cough attributes | 323 (31.18) |
|  | Dizziness attributes | 180 (17.37) |
|  | Chest pain attributes | 48 (4.63) |
|  | Heartbeat and palpitation attributes | 37 (3.57) |
|  | Bleeding attributes | 0 (0.00) |
|  | Headache attributes | 23 (2.22) |
| **Lifestyle attributes, n (%)** | | |
|  | Fluid and salt Intake | 3605 (72.22) |
|  | Smoking and alcohol | 1387 (27.78) |
| **Vitals attributes, n (%)** | | |
|  | Blood pressure | 28,358 (62.36) |
|  | Weight | 17,116 (37.64) |
| **Speakers, n (%)** | | |
|  | Nurse telecarer | 87,476 (54.59) |
|  | Patient | 58,833 (36.71) |
|  | Caregiver | 13,571 (8.47) |
|  | Others | 371 (0.23) |
