# Supplementary material for: Characterization of Telecare Conversations on Lifestyle Management and Their Relation to Health Care Utilization for Patients with Heart Failure: Mixed Methods Study
Source: J Med Internet Res. 2024 Oct 30;26:e46983. doi: 10.2196/46983 (PMC11561433; doi:10.2196/46983)
Supplement: Multimedia Appendix 3 [file jmir_v26i1e46983_app3.docx]

**Multimedia Appendix 3**

Multimedia Appendix 3 (Table). Examples of dialogue acts annotated in the dataset.

| **Dialogue acts** | | **Examples** |
| --- | --- | --- |
| **Exchanging information** | | |
|  | request-inform | “(orh) okay okay then you you you got jab insulin or not?”  “so far medicine giving you any side effects or not? any, any issues?” |
|  | inform | “(ah), so anything that you think is very salty (ah), your water retention will get worse.”  “digoxin I think it's blue color tablet”  “I eat little bit only”  “(ah) but just now I come to work I forgot to take (yah)”  “I stop my what you call the (ah) urine ((that one))” |
| **Understanding information** | | |
|  | acknowledge | “okay, 4 cups (ah)”, “yes okay”, “(mm) (mm)”, “I see”, “right. right” |
|  | request-clarification | “you cook with what things?”  “sorry, how many times a day? Can you repeat?” |
|  | request-confirmation | “(ah) one one litre is it?”  “so you took the medicine or you didn't take?” |
| **Performing action** | | |
|  | request-action | “so remember the fluid, don't take so much salt (hor)”  “so continue to take your (uh) water pills (ah), furosemide, forty milligrams this morning, (hor)?”  “okay today you stop your half tablet of lasix (ah)” |
|  | accept-action-implicit | “okay”, “(yah)”, “(mm)”, “I know I know” |
|  | accept-action-explicit | “okay. I will do that tomorrow.” |
|  | reject-action-implicit | Nurse telecarer: “so remember to follow the new dosage”  Patient: “(um) but I feel much better now” |
|  | reject-action-explicit | “no, I don’t have time to check” |
| **Evaluation of health condition** | | |
|  | evaluate | “so you must be very stressed (hor)”  “(mm). so your weight, everything is the same (la). there's no increase (ah).” |
|  | evaluate-positive | “(orh). good, keep it up”  “(yah) at this point you can laugh that that is good (lor) (mm)” |
|  | evaluate-negative | “(yah) no wonder I could could tell that you don't sound (er) so well.”  “I think so (la) I very worried” |
| **Social emotional** | | “"aiya" just just go and pray (la) (hor) everything just pray for good health (la)”  “thank God (lah) my husband help me” |
| **Incomplete dialogue act** | | |
|  | back-channel | “(uh-huh)”, “(yah)”, “(huh)”, “(mm-hmm)”, “(orh)”, “(ah)”, “(oh)”. |
|  | fragment | “so (ah)”, “but”, “I think the... (yah)”, “okay actually”, “(yah) because (yah)”. |
|  | stall | “what did you eat- (eh) no then how’s your fluid intake?” |
| **Others** | | “you know what I'm saying?”  “let me tell you” |
