# Supplementary material for: Characterization of Telecare Conversations on Lifestyle Management and Their Relation to Health Care Utilization for Patients with Heart Failure: Mixed Methods Study
Source: J Med Internet Res. 2024 Oct 30;26:e46983. doi: 10.2196/46983 (PMC11561433; doi:10.2196/46983)
Supplement: Multimedia Appendix 5 [file jmir_v26i1e46983_app5.docx]

**Multimedia Appendix 5**

Multimedia Appendix 5 (Table)*.* Characteristics of Full cohort (50 patients with HF^a^), HFDM^b^ cohort (25 patients with both HF^a^ and T2DM^c^), and expert evaluation cohort (11 patients with both HF^a^ and T2DM^c^, a subset of the HFDM^b^ cohort).

|  | | | Full cohort (N=50)^d^ | HFDM^b^ cohort (n=25)^d^ | Expert evaluation cohort (n=11)^d^ |
| --- | --- | --- | --- | --- | --- |
| **Charlson Comorbidity Index (CCI), n (%)** | | |  |  |  |
|  | | 0 | 16 (32) | 5 (20) | 3 (27) |
|  | | 1-4 | 21 (42) | 11 (44) | 3 (27) |
|  | | 5-9 | 8 (16) | 4 (16) | 3 (27) |
|  | | >=10 | 5 (10) | 5 (20) | 2 (18) |
| **Medication treatment class^e^, n (%)** | | | | | |
|  | Beta-adrenergic blocking agents | | 46 (92) | 22 (88) | 9 (82) |
|  | Renin-angiotensin-aldosterone system inhibitors | | 1 (2) | 0 (0) | 0 (0) |
|  | Angiotensin-converting enzyme inhibitors | | 33 (66) | 17 (68) | 6 (55) |
|  | Angiotensin II receptor antagonists | | 21 (42) | 12 (48) | 5 (45) |
| **NYHA class, n (%)** | | | | | |
|  | Class 1 | | 12 (24) | 7 (28) | 4 (36) |
|  | Class 2 | | 21 (42) | 9 (36) | 4 (36) |
|  | Class 3 | | 8 (16) | 3 (12) | 0 (0) |
|  | Class 4 | | 5 (10) | 4 (16) | 2 (18) |

^a^HF: heart failure.

^b^HFDM: patients with both heart failure and type 2 diabetes mellitus.

^c^T2DM: type 2 diabetes mellitus.

^d^For some indicators, data may not be available for all patients in the cohort.

^e^Proportions of patients with specific medication classes may not be mutually exclusive.
