# Supplementary material for: Characterization of Telecare Conversations on Lifestyle Management and Their Relation to Health Care Utilization for Patients with Heart Failure: Mixed Methods Study
Source: J Med Internet Res. 2024 Oct 30;26:e46983. doi: 10.2196/46983 (PMC11561433; doi:10.2196/46983)
Supplement: Multimedia Appendix 6 [file jmir_v26i1e46983_app6.docx]

## **Multimedia Appendix 6**

Patient: *I'm having a bad cough (ah)* </inform>

Telecarer: *bad cough (hah)* </request-confirmation>

Patient: *(mm)* </acknowledge>

Telecarer: *(oh) are you on water restriction or not (ah)?* </request-inform>

Patient: *(yah)(yah)* </inform>

Telecarer: *(ah) one one litre is it?* </request-inform>

Patient: *(yah) one point two* </inform>

Telecarer: *one point two (hor)* </request-confirmation>

Patient: *(ah)(yah)* </inform>

Telecarer: *so remember the fluid, don't take so much salt (hor)* </request-action>

Patient: *(oh) okay* </accept-action-implicit>

Telecarer: *(hor) and as worried that you (ah) "kena"* [suffer something unpleasant] *swell, water retention (ah) that's why (lah)* </inform>

Patient: *(mm) okay* </acknowledge>

[P05, male, 50-59 years old, Chinese]

Multimedia Appendix 6 (Textbox). Excerpt of conversation on lifestyle management between patient P05 and nurse telecarer N02.
