# Supplementary material for: Characterization of Telecare Conversations on Lifestyle Management and Their Relation to Health Care Utilization for Patients with Heart Failure: Mixed Methods Study
Source: J Med Internet Res. 2024 Oct 30;26:e46983. doi: 10.2196/46983 (PMC11561433; doi:10.2196/46983)
Supplement: Multimedia Appendix 7 [file jmir_v26i1e46983_app7.docx]

## **Multimedia Appendix 7**

Multimedia Appendix 7 (Textbox). Excerpt of conversation on lifestyle management between patient P03’s spouse and nurse telecarer N06.

Spouse: *(er) there's some swelling on the feet.* </inform>

Telecarer: *(uh-oh). (oh) dear.* </evaluate-negative> *but the fluid part okay or not. the the the fluid okay or not?* </request-inform> […]

Spouse: *flu- fluid (())* </stall> *he sometimes don't know (er)... don't want to listen.* </inform> […]

Spouse: *I become very firm with him.* </inform>

Telecarer: *(orh) very firm (ah).* </acknowledge> *(oh) dear.* </evaluate-negative>

Spouse: *because I say (ah) I say this cannot go on.* </inform>

Telecarer: *{laughter}. (oh) (wah). really very stressful on your part (ah).* </social-emotional>

Spouse: *(ah) what to do* </social-emotional>

Telecarer: *(yah) what to do? you are you are (()) wife (ah).* </social-emotional>

Spouse: *so what to do?* </social-emotional> *because--* </stall> *and he takes anything he want put ice on it*. </inform> […]

Telecarer: *(mm). "aiyo".* </evaluate-negative>

Telecarer: *(er) need to watch out all extra you know. extra he didn't think (oh) icy (ah). love icy drink. (mm).* </request-action>

Spouse: *so I told him I said I said no more (()) for you.* </inform> *(yah). (yah). (yah).* </accept-action-explicit> […]

Telecarer: […] "*aiya" I think Mr. [name_patient] got tired of the of the strict fluid control I think.* </inform>

Spouse: *(yah). (()).* </back-channel>

Telecarer: *probably got tired already (la).* </inform>

Spouse: *what to do.* </social-emotional>

[P03, male, 80-89 years old, Indian]
