# Supplementary material for: Characterization of Telecare Conversations on Lifestyle Management and Their Relation to Health Care Utilization for Patients with Heart Failure: Mixed Methods Study
Source: J Med Internet Res. 2024 Oct 30;26:e46983. doi: 10.2196/46983 (PMC11561433; doi:10.2196/46983)
Supplement: Multimedia Appendix 8 [file jmir_v26i1e46983_app8.docx]

## **Multimedia Appendix 8**

Multimedia Appendix 8 (Table). Comparison of proportions of lifestyle-focused calls (n=100) and of calls with lifestyle management content (not focused; n=138) that include mentions of symptom attributes.

|  | Proportion of lifestyle-focused calls with mention of symptom attributes (n=100) | Proportion of calls with lifestyle management content (not focused) with mention of symptom attributes (n=138) |
| --- | --- | --- |
| Any symptom, n (%) | 53 (53%) | 81 (59%) |
| Swelling, n (%) | 19 (19%) | 28 (20%) |
| Breathlessness, n (%) | 9 (9%) | 18 (13%) |
| Cough, n (%) | 28 (28%) | 53 (38%) |
| Dizziness, n (%) | 11 (11%) | 20 (14%) |
| Heartbeat, n (%) | 4 (4%) | 6 (4%) |
| Chest pain, n (%) | 2 (2%) | 6 (4%) |
| Headache, n (%) | 1 (1%) | 2 (1%) |
