# Supplementary material for: Characterization of Telecare Conversations on Lifestyle Management and Their Relation to Health Care Utilization for Patients with Heart Failure: Mixed Methods Study
Source: J Med Internet Res. 2024 Oct 30;26:e46983. doi: 10.2196/46983 (PMC11561433; doi:10.2196/46983)
Supplement: Multimedia Appendix 9 [file jmir_v26i1e46983_app9.docx]

## **Multimedia Appendix 9**

Multimedia Appendix 9 (Table). Comparative analysis of average length of stay (LOS) for all-cause inpatient admissions and number of all-cause inpatient admissions between patients with at least 1 lifestyle-focused call (n=30) and patients with no lifestyle-focused calls (n=20).

|  | Patients with at least 1 lifestyle-focused call (n=30) | | Patients with no lifestyle-focused call (n=20) | | Cohen *d* |
| --- | --- | --- | --- | --- | --- |
|  | Mean (SD) | Median (IQR) | Mean (SD) | Median (IQR) |  |
| Average length of stay (LOS) | 6.1 (2.3) | 6.3 (4.5-6.8) | 7.3 (2.9) | 6.9 (5.0-9.3) | -0.62 |
| Number of inpatient admissions | 3.2 (2.1) | 3.0 (2.0-4.0) | 4.0 (1.4) | 4.0 (1.0-5.0) | -0.21 |
